# Supplementary material for: Identification of the Unwinding Region in the Clostridioides difficile Chromosomal Origin of Replication
Source: Front Microbiol. 2020 Oct 2;11:581401. doi: 10.3389/fmicb.2020.581401 (PMC7561715; doi:10.3389/fmicb.2020.581401)
Supplement: Supplementary file 1 [file Data_Sheet_1.PDF]

Supplementary material

Identification of the unwinding region in the *Clostridioides difficile* chromosomal origin of replication

Ana M. Oliveira Paiva[1,2], Erika van Eijk [1], Annemieke H. Friggen[1], Christoph Weigel[3], Wiep Klaas Smits[1,2]*

1. Supplementary Data

Pattern search:
NTATCCACA
TNATCCACA
TTANCCACA
TTATCNACA
TTATCCNCA
TTATCCANA
TTATCCACN
TTNTCCACA
TGTGGATAN
TGTGGATNA
TGTGGNTAA
TGTNGATAA
TGNGGATAA
TNTGGATAA
NGTGGATAA
TGTGGANAA
TTWTNCACA
TGTGNAWAA
NTWTNCACA
TGTGNAWAN
TNWTNCACA
TGTGNAWNA
TTWNNCACA
TGTGNNWAA
TTWTNNACA
TGTNNAWAA
TTWTNCNCA
TGNGNAWAA
TTWTNCANA
TNTGNAWAA
TTWTNCACN
NGTGNAWAA

2. Supplementary Figures

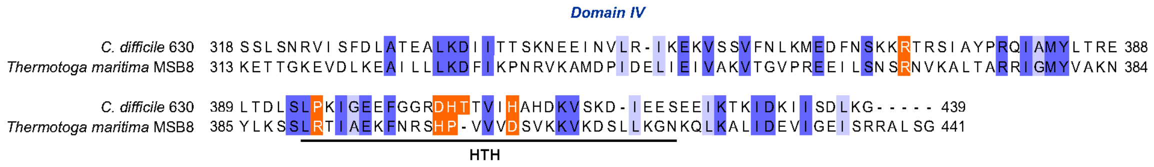

Figure S1. **Alignment of domain IV of the *C. difficile* and *Thermotoga maritima* DnaA protein**. Residues are colored according to sequence identity conservation **using** blue shading (dark blue more conserved), as analysed in JalView, as for Figure 1. Residues involved in specific contacts with the 9-mer DnaA box sequence are indicated in orange. It is clear that the majority of these residues are not conserved between the two species, except *C. difficile* R370/*T. maritima* R366.

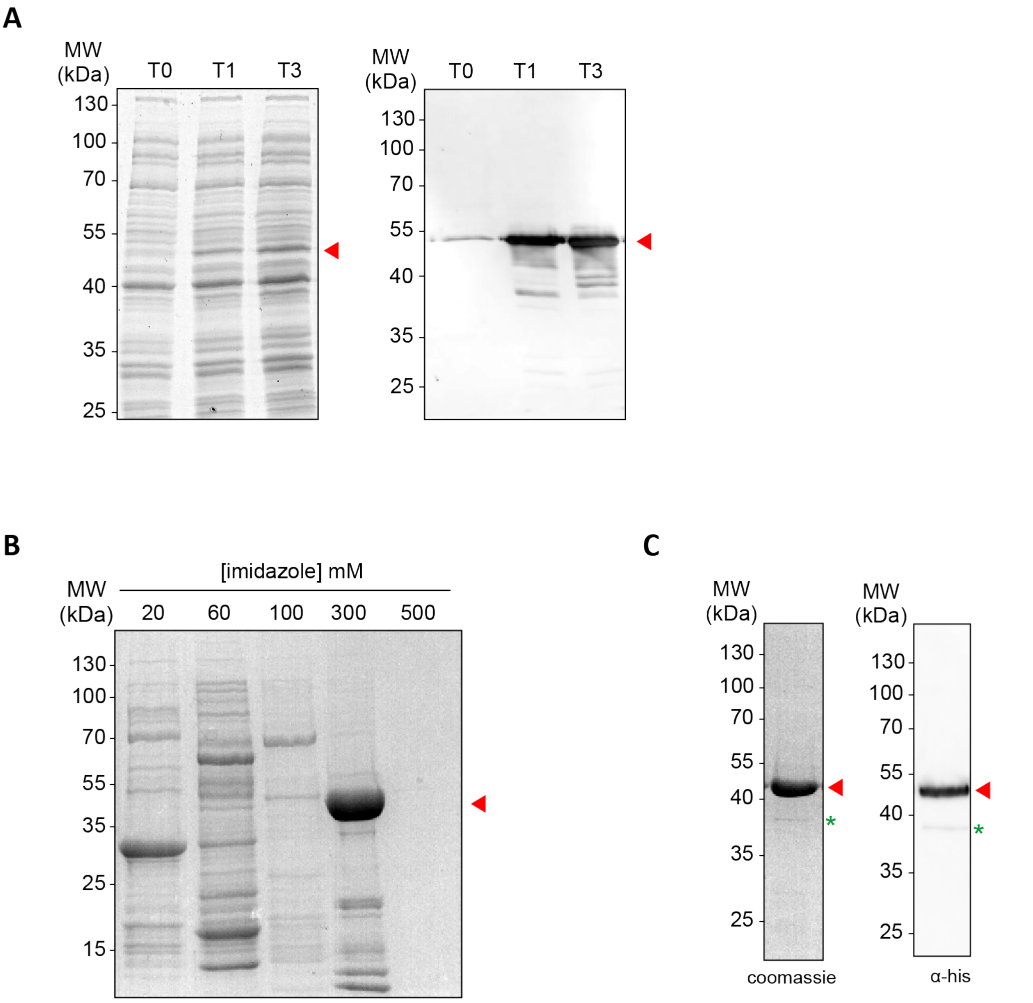

Figure S2. Expression and purification of *C. difficile* DnaA-6xHis protein. A) *E. coli* expressing DnaA-6xHis cells were induced with 1 mM IPTG. Optical density-normalized samples before induction (T0), after 1 hour of induction (T1) and 3 hours of induction (T3) were resolved by 12% SDS-PAGE and immunoblotted with anti-his antibody. Induced DnaA is observed with the approximate molecular weight of 51 kDa (red arrow). Possible breakdown product is observed (blue arrow). **B)**. Samples of DnaA-6xHis HisTrap purification from the elution fraction 2 at binding buffer with different imidazole concentrations (20, 60, 100, 300 and 500 mM) were separated by 12% SDS– PAGE and stained with Coomassie brilliant blue. DnaA-6xHis is observed with an approximate molecular weight of 51 kDa (red arrow), and eluted in Binding buffer supplemented with >300 mM imidazole. **C)** Confirmation of size-exclusion fraction containing the *C. difficile* DnaA-6xHis and further used for analysis after protein purification resolved by 12% SDS-PAGE (Coomassie staining) and immunoblotted with anti-his antibody. DnaA-6xHis is observed with the approximate molecular weight of ~51 kDa (red arrow). Possible minor breakdown products are observed (green asterisk).

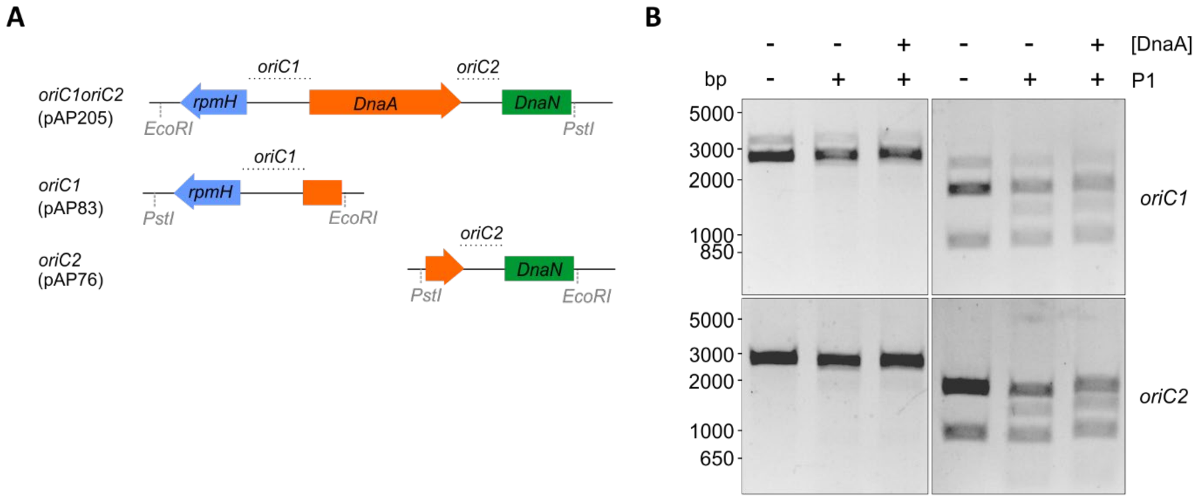

Figure S3. P1 nuclease assay of the individual *C. difficile oriC regions*. A) Representation of the *oriC* regions present in the used vectors for P1 nuclease assay, *oriC1oriC2* (pAP205), *oriC1*-(pAP83) and *oriC2* (pAP76)-containing vectors. The predicted *oriC* regions (dotted lines) and included genes are represented, *rpmH* (blue), *dnaA* (orange), and *dnaN* (green). P1 nuclease assay of pAP83 (*oriC1*, upper panel) and pAP76 (*oriC2*, lower panel). Digestion of the vector with the restriction enzymes BglII (left panel) or NotI (right panel). Digestion of the vectors with the restriction enzymes (lanes 1-3). Treatment of the fragments with P1 nuclease only (lane 2) and incubated with 0.14 µM of *C. difficile* DnaA-6xHis protein (lane 3). Higher DnaA-6xHis were tested with same profile (data not shown). The DNA fragments were separated in a 1% agarose gel and analyzed with ethidium bromide staining. Spontaneous unwinding is observed and no DnaA-dependent unwinding is detected.

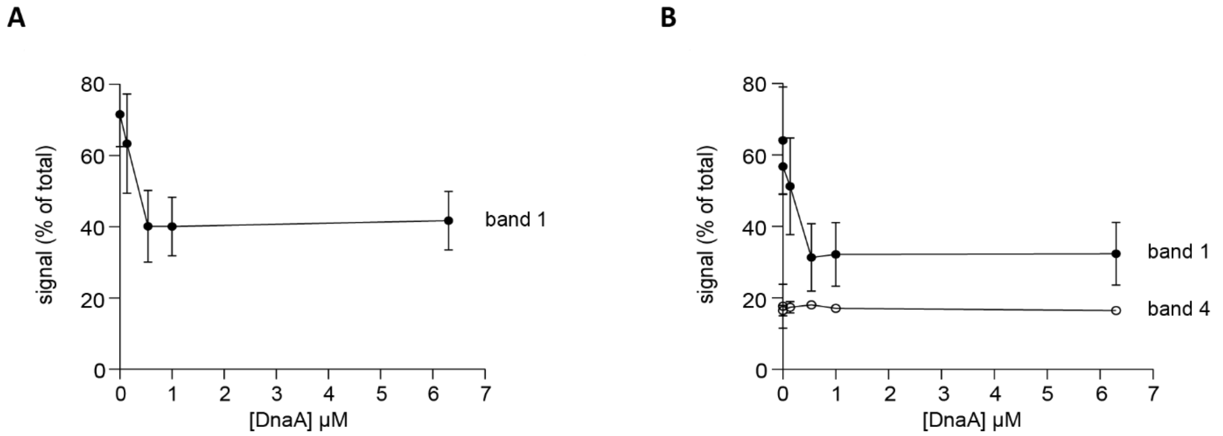

Figure S4. Quantification of the P1-independent bands. Data presented here are complementary to that of Figure 4C and 4D in the main body of the manuscript. Quantification was performed using ImageJ, and signals were normalized to the total signal in a lane. **A**. Results for P1/BglII digested vector. Shown is the signal (black circles) for the upper band of the gel (Figure 4B, upper panel). **B**. Results for the P1/NotI digested vector. Shown is the quantification of the signal of the upper (black circles) and lower (open circles) bands of the gel (Figure 4B, lower panel). Error bars indicate the standard deviation of the mean of n=3 independent experiments.

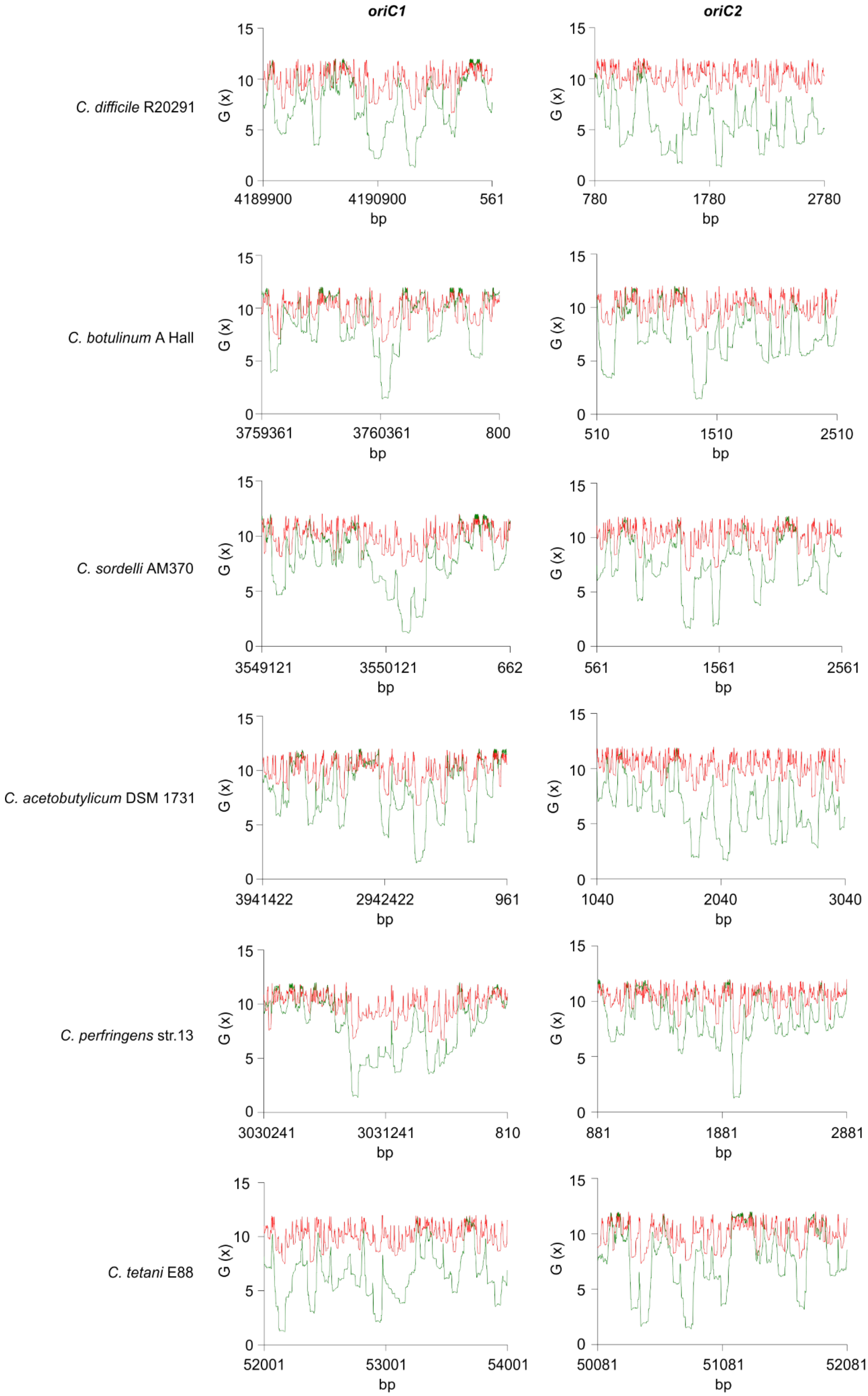
oriC1
oriC2
C. difficile R20291
C. botulinum A Hall
C. sordelli AM370
C. acetobutylicum DSM 1731
C. perfringens str.13
C. tetani E88
G (x)
bp
15
10
5
0
4189900
4190900
561
780
1780
2780
3759361
3760361
800
510
1510
2510
3549121
3550121
662
561
1561
2561
3941422
2942422
961
1040
2040
3040
3030241
3031241
810
881
1881
2881
52001
53001
54001
50081
51081
52081

Figure S5. SIDD analysis different clostridia. Analyis of 2.0 kb fragments comprising *oriC1* and *oriC2* in *C. difficile* R20291, *C. botulinum A* Hall, *C. sordelli* AM370, *C. acetobutylicum* DSM 1731, *C. perfringens* str.13, *C. tetani* E88 (see Table 1 in the main body of the manuscript). Nucleotide positioning is indicated. Predicted free energies G(x) for duplex destabilization at a superhelical density of σ = -0.06 (green) or σ = -0.04 (red).

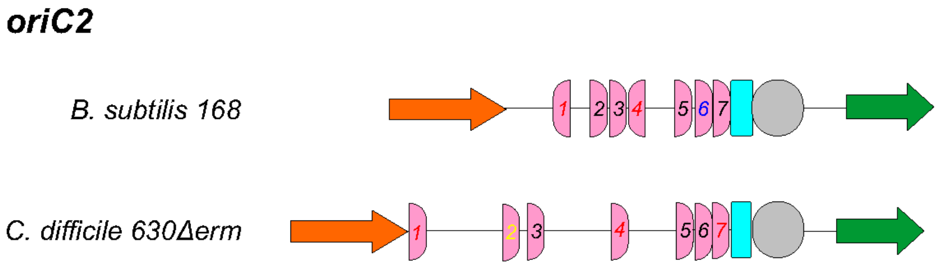

Fig. S6. Comparison of the *B. subtilis* and *C. difficile oriC2*. Representation of the *oriC2* region (intergenic region between *dnaA* and *dnaN*) of *B. subtilis* and *C. difficile* chromosome. The *dnaA* and *dnaN* genes are represented by orange and green arrows, respectively. The DUE is represented by a grey circle. DnaA-trio sequences are shown in light blue boxes. DnaA-boxes are indicated by pink boxes and orientation on the leading (right) and lagging strand (left) are shown. DnaA boxes are numbered according to the *B. subtilis* nomenclature (Richardson, 2019), with numbers in blue (no mismatch from the TTATCCACA sequence, red (1 mismatch), black (2 mismatches) or yellow (3 mismatches). See Material and Methods for detailed information. Alignment of the represented chromosomal regions is based on the location of the DnaA-trio.
